# Umbilical cord mesenchymal stem cell exosomal miR-143-3p delays endothelial cell senescence through targeting COX-2

**DOI:** 10.1371/journal.pone.0327173

**Published:** 2025-07-11

**Authors:** Zhi-peng Yang, Shui-hong Lu, Yan-hong Pan, Zhao-fu Liao, Yi-tuan Xie, Heng Li, Yu-lan Zhou, Zhen-can Shi, Yun-fei Qu, Zhu-guo Wu, Chongxiang Xiong, Xing-dong Xiong

**Affiliations:** 1 Dongguan Key Laboratory of Aging and Anti-Aging, Guangdong Provincial Key Laboratory of Medical Immunology and Molecular Diagnostics, Cardiovascular Center, The First Dongguan Affiliated Hospital, Guangdong Medical University, Dongguan, P.R. China; 2 School of Medical Technology, The Second Affiliated Hospital of Guangdong Medical University, Guangdong Medical University, Dongguan, P.R. China; 3 Department of Cerebrovascular Disease, Huizhou First People’s Hospital, Huizhou, P.R. China; 4 Department of Cardiovascularology, Dongguan Tungwah Hospital, Dongguan, P.R. China; 5 Department of Nephrology, The First Dongguan Affiliated Hospital of Guangdong Medical University, Dongguan, P.R. China; Universita degli Studi di Perugia, ITALY

## Abstract

Senescence of vascular endothelial cells leads to endothelial dysfunction and exacerbates atherosclerosis. In this study, we presented evidence that exosomes derived from human umbilical cord mesenchymal stem cells (hucMSC-Exos) could delay endothelial cell senescence, promote endothelial cell proliferation, and enhance angiogenic activity in vitro. The miRNA profiling analysis revealed a high expression of miR-143-3p in hucMSC-Exos, which was further upregulated in endothelial cells treated with hucMSC-Exos. Silencing miR-143-3p induced endothelial cell senescence, as evidenced by increased senescence-associated *β*-galactosidase activity, reduced cell proliferation, and inhibited tubular formation; conversely, overexpression of miR-143-3p exhibited opposite effects. Moreover, we found that miR-143-3p directly targeted Cyclooxygenase-2 (COX-2) and suppressed its translation, thus delaying endothelial cell senescence. These results suggested that hucMSC-Exos can delay endothelial cell senescence by transferring miR-143-3p. In summary, our data demonstrated the potential of hucMSC-Exos as an intervention against vascular aging.

## Introduction

Aging is an independent risk factor for cardiovascular disease, which is the leading cause of global mortality [1]. The vascular endothelium, composed of a monolayer of endothelial cells, serves as a barrier between the vascular wall and the circulation while regulating vascular tone and maintaining vascular homeostasis [2]. Evidence suggests that endothelial senescence impairs endothelium-dependent dilation, angiogenesis, and barrier function, potentially triggering the onset of numerous age-related diseases [3]. Our previous study demonstrated that endothelial senescence was a pivotal event in the progression of atherosclerosis [4]. However, the potential interventions for delaying endothelial senescence to prevent age-related vascular diseases remain unclear.

Mesenchymal stem cells (MSCs) are characterized by their robust self-renewal properties and their capacity to differentiate into various cell types, which has garnered significant interest due to their substantial therapeutic potential [5]. Research has demonstrated the safety and potential efficacy of stem cells in treating multiple diseases [6]; however, the low retention and survival rates of delivered stem cells hinder their clinical application [7]. Given the mounting evidence, it is increasingly clear that the therapeutic benefits of MSCs are achieved through a paracrine mechanism, in which exosomes play a vital role [8]. Exosomes are vesicle-like bodies with a bilayer membrane structure ranging from 30 to 150 nm that are released through fusion with the cell membrane [9]. Exosomes act as intercellular messengers by transferring biomolecules such as nucleic acids, proteins, and lipids, which in turn affect the activities of recipient cells [10]. Similar to their parent cells, MSC-derived exosomes improve tissue repair and regeneration by promoting angiogenesis or modulating immune responses [11,12]. Accordingly, MSC-derived exosomes represent a promising acellular alternative for stem cell therapy owing to their striking advantages, including low immunogenicity, easy storability and high biosafety.

Human umbilical cord mesenchymal stem cells (hucMSCs) offer several benefits over MSCs derived from other sources, including non-invasive collection procedures, low immunogenicity, and no ethical controversy, which makes hucMSCs an ideal candidate for preparing therapeutic exosomes [13]. Recently, exosomes derived from hucMSCs (hucMSC-Exos) have demonstrated potential in anti-inflammation and injury repair [14,15]. Nonetheless, the effects and mechanisms of hucMSC-Exos on senescence remain largely unknown, particularly on endothelial senescence.

This study revealed that hucMSC-Exos were capable to alleviate endothelial cell senescence, stimulate cell proliferation, and enhance angiogenic activity in vitro. Additionally, we found that miR-143-3p was abundant in hucMSC-Exos, and played a key role in delaying endothelial senescence by inhibiting Cyclooxygenase-2 (COX-2).

## Materials and methods

### Cell culture

To generate hucMSC-Exos, HucMSCs were maintained in α-MEM (Gibco, USA) supplemented with 5% UltraGRO serum replacement (AventaCell BioMedical, USA). HucMSCs in passages 3–5 were used in subsequent experiments. Human coronary artery endothelial cells (HCAECs) were purchased from ScienCell Research Laboratories (ScienCell, USA). To ensure optimal conditions for their growth, these cells were cultured using a specialized endothelial cell medium (ECM). This medium was fortified with 5% fetal bovine serum, which provides essential nutrients and growth factors, along with 1% endothelial cell growth supplement to further promote cell proliferation. The culture process took place in a humidified setting at 37°C, where a carbon dioxide concentration of 5% was consistently upheld. The method for defining “senescent cells” and “proliferating cells” by calculating population doubling level (PDL) is described in previous study [16].

### Exosome isolation

HucMSC-Exos were extracted from the conditioned medium via a process of ultra-differential centrifugation. When MSCs reached 80%−90% confluence, the supernatants containing hucMSC-Exos were harvested. For the removal of dead cells and cellular debris, the initial step involved centrifuging the conditioned medium at two different speeds: 300 × g and then at 2,000 × g, with each centrifugation lasting for 10 min. Then, to eliminate larger microvesicles, the supernatant was subject to a further centrifugation at a higher speed of 10,000 × g for 30 min. The leftover supernatant was then processed through ultracentrifugation at 100,000 × g for 70 min to collect the pelleted exosomes. After this, the sample was resuspended in PBS and centrifuged again at 100,000 × g for 70 min. Ultimately, the resulting precipitates were resuspended in PBS and passed through a 0.22 μm filter, which further removed the residual large particles. The protein concentration was determined by BCA assay kit (Beyotime, China).

### Transmission electron microscopy

During sample preparation, 10μl of hucMSC-Exos was applied onto a copper grid that had been coated with carbon, allowing it to rest for 2 min. Following this, excess liquid was removed using filter paper. Next, the samples underwent staining in a 2% uranyl acetate for 1 min to improve their visibility under the microscope and were then allowed to dry. Ultimately, the morphological structure of hucMSC-Exos was visualized by JEM-1200 EX Electron Microscope (JEOL, Japan).

### Nanoparticle tracking analysis

Flow Nano Analyzer ((NanoFCM, China) was utilized to measure size distribution and granular concentration of hucMSC-Exos. The camera captured the light scattered by the exosomes when illuminated by a laser, recording a video file of hucMSC-Exos undergoing Brownian motion. Particles were tracked, quantitated, and their sizes determined using NTA software.

### Western blot analysis

RIPA buffer with protease inhibitors was utilized to prepare cell lysates. Subsequently, the lysates were loaded on 12% SDS-PAGE gels, where the proteins were separated via electrophoresis. After completed electrophoresis, the proteins were transferred to the PVDF membranes (Merck Millipore, USA). Next, the membranes were blocked for 1h with 5% skim milk in TBST and then exposed to primary antibodies against CD63, CD81, TSG101, Calnexin, p16 and *β*‐actin (Cell Signaling Technology, USA). To ensure optimal binding between the antibodies and their respective target proteins, an overnight incubation was conducted at a cold temperature of 4°C. Following additional incubation with the HRP-linked secondary antibody (Beyotime, China) at room temperature for 2 hours, the bands were visualized using Immobilon Western Chemiluminescent HRP Substrate (Merck Millipore, USA). Later, band intensities were quantified with the ImageJ software.

### Internalization of exosomes

Endothelial cells were labeled with the fluorescent lipophilic tracer Vybrant Dil dye (Invitrogen, USA). After the introduction of Dil solution into the culture medium, the mixture was incubated with endothelial cells under 5% humidified CO_2_ for 15 min and washed three times in PBS. Later, PKH67 (Sigma, USA) was added to the hucMSC-Exos suspension and incubated at room temperature away from light for 15 min. PKH67-labeled exosomes were subjected to centrifugation at 100,000 × g for 70 min to remove excess dye, subsequently resuspending the resultant precipitate in PBS. These labeled exosomes were co-cultured with the recipient cells and incubated at 37°C for 4 h. After triple washes in PBS, the cells were fixed using 4% paraformaldehyde for 15 min, followed by a 5-min DAPI staining. Ultimately, a fluorescence microscope was employed to capture images of the samples for further analysis.

### Exosome treatment

Cells for hucMSC-Exos treatment were cultured in ECM with 10% exosome-depleted FBS. 20 μg/ml hucMSC-Exos were then added and co-cultured with endothelial cells for 5 days. The cells were subcultured when they reached an 80–90% confluence and the media containing exosomes was replaced.

### Senescence-associated *β*-galactosidase staining

Evaluation of cellular senescence was evaluated through the use of an SA-*β*-gal staining kit (Beyotime, China) to measure *β*-galactosidase activity. Concisely, cells were rinsed with PBS, followed by fixation in a fixative solution at room temperature for 15 min, Subsequently, cells were placed in a newly prepared staining solution and incubated overnight at 37°C. The proportion of blue-stained senescent cells was calculated by counting stained cells across multiple randomly chosen microscopic fields under Eclipse TS100 Inverted Microscope (Nikon Corporation, Japan).

### BrdU incorporation assay

The assessment of cell proliferation was conducted using a BrdU incorporation assay kit. Initially, the cells were incubated in a medium supplemented with 40 μM BrdU for 1 h. Fixed cells with ethanol underwent permeabilization using with 0.05% trypsin and were blocked with 0.5% BSA at room temperature for 1 h. The detection of incorporated BrdU was achieved by the incubation with an anti-BrdU mouse monoclonal antibody (CST, USA) for 1 h at 37°C, followed by an Alexa Fluor 488-conjugated anti-mouse immunoglobulin G (IgG) antibody (CST, USA) for 1 h at 37°C. During imaging of BrdU-positive cells, Invitrogen EVOS FL Auto Cell Imaging System (Thermo Fisher Scientific, USA) was used with magnification set to 100 × .

### Tube formation assay

To assess tube formation, endothelial cells were added to a 96-well culture plate that had been pre-coated with 100μl Matrigel at a density of 1 × 10^4^ cells per well. Following 6h of incubation period, tube formation was observed and photographed under an inverted microscope at 100 × magnification (Eclipse TS100, Japan). Total tube length were subsequently quantified utilizing ImageJ software.

### Transwell assay

A 24-well plate transwell chamber (Corning, USA) was used for the cell migration assay. 200 μl serum-free medium with hucMSC-Exos containing 4 × 10^4^ endothelial cells was added to the upper chamber, and the lower compartment was filled with 700 μl 10% FBS medium. Following a 24-hour incubation period, cells located above the filter that had not migrated were carefully removed with a cotton swab. Then, the filters were fixed with 70% ethanol and followed by crystal violet staining. Migratory cells were quantified in three microscopic fields per chamber at 100 × magnification.

### RNA isolation and qPCR

For RNA isolation, the samples were lysed using TRIzol® reagent (Invitrogen, USA). The cDNA of the miRNA was generated using a miRNA 1st Strand cDNA Synthesis Kit (Vazyme, China), and subsequent qPCR was conducted using miRNA Universal SYBR qPCR MasterMix (Vazyme, China). The relative expression levels was calculated using the 2^-ΔΔCt^ comparative threshold method, with U6 serving as an internal reference for quantification of miRNA. The specific primers used are listed in S1 Table in S1 File.

### MicroRNA sequencing and microRNA-targeted genes prediction

Small RNAs were sequenced by the Novogene Corporation (Beijing, China). Total RNA extracted from the exosomes was used for library construction with the TruSeq® Small RNA Sample Prep Kit (Illumina, USA). The miRNA libraries were sequenced on a HiSeq TM 2500 platform (Illumina, USA) with 50 bp single-end reads strategy. Clean reads were obtained by removing reads with adapters, reads with more than 10% N (N means that the base type cannot be determined), reads containing poly A/T/G/C stretches, reads shorter than 18nt and low-quality raw reads. After filtration, clean reads were compared with miRNA databases (miRbase 20.0) to annotate the mature small RNA sequences and known miRNA precursors by Bowtie. The target genes of miR-143-3p were predicted by the online software Targetscan (www.targetscan.org/). KEGG pathway enrichment and GO functional enrichment were respectively implemented by the KEGG database (http://www.genome.jp/kegg/) and GO database (http://geneontology.org).

### Cell transfection

The miRNA mimics and inhibitors, along with corresponding negative control for miR-143-3p were obtained from Gemma Pharmaceutical Technology (Shanghai, China). The utilized sequences are shown in S1 Table in S1 File. The transfection process was performed with Lipofectamine RNAiMax Reagent (Thermo Fisher Scientific, USA) following the guidelines provided by the manufacturer.

### Statistical analysis

Statistical analyses were performed with SPSS 19.0 (IBM, USA) and GraphPad Prism 8.0 (GraphPad Software, USA). Data are shown as mean ± standard deviation (SD) or SEM. Statistical significance was assessed by an independent samples *t*-test and one-way ANOVA with least significant difference (LSD) test, with a value of *p* < 0.05 considered statistically significant.

## Results

### Characterization and internalization of exosomes derived from hucMSCs

**Fig 1:**
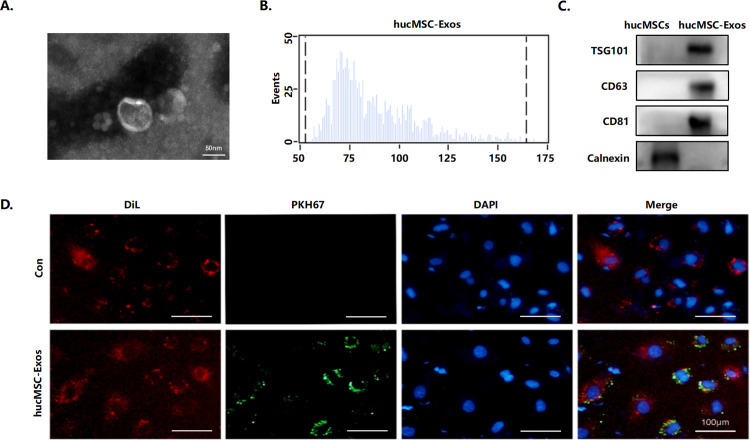
Characterization and internalization of hucMSC-Exos derived from hucMSCs. (A) Transmission electron microscopy images of hucMSC-Exos. Scale bars = 50 nm. (B) Nanoparticle tracking analysis of the size distribution of hucMSC-Exos. (C) Exosome marker proteins (TSG101, CD63, CD81 and calnexin) in hucMSC-Exos were analyzed by Western blot. (D) Uptake of hucMSC-Exos by HUVECs was determined by fluorescence microscope. Dil-HUVECs were incubated with 20 µg/mL PKH67-labeled hucMSC-Exos for 4 h. Scale bars = 100 μm.

HucMSCs were identified by surface marker expression and multi-lineage differentiation ability. HucMSCs exhibited positive expression of MSC markers, while lacking expression of hematopoietic markers. Furthermore, these cells exhibited the capability to differentiate into osteoblasts and adipocytes (S1 Fig in S1 File). Exosomes were isolated from the supernatant of hucMSCs via ultracentrifugation and characterized by several techniques. Transmission electron microscopy (TEM) analysis showed that hucMSC-Exos displayed a characteristic cup-shaped morphology (Fig 1A). Nanoparticle tracking analysis (NTA) results demonstrated that the size of hucMSC-Exos was between 50–175 nm, with an average particle size of 80 nm, matching the typical size distribution of exosomes (Fig 1B). Common exosome protein markers (CD63, CD81 and TSG101) were present in hucMSC-Exos; the negative protein marker (calnexin) was not found in hucMSC-Exos compared to cell lysate when analyzed by western blot (Fig 1C). HUVECs were used as a cellular model to investigate the internalization ability of hucMSC-Exos by endothelial cells. Under fluorescence microscope, hucMSC-Exos labeled with PKH67 were added to Dil-labeled endothelial cells, showing that hucMSC-Exos could be successfully taken up by endothelial cells (Fig 1D). The above results indicated that the extracted hucMSC-Exos met the identification criteria for exosomes.

### hucMSC-Exos delayed senescence, promoted proliferation, and increased angiogenic activity of endothelial cells

**Fig 2:**
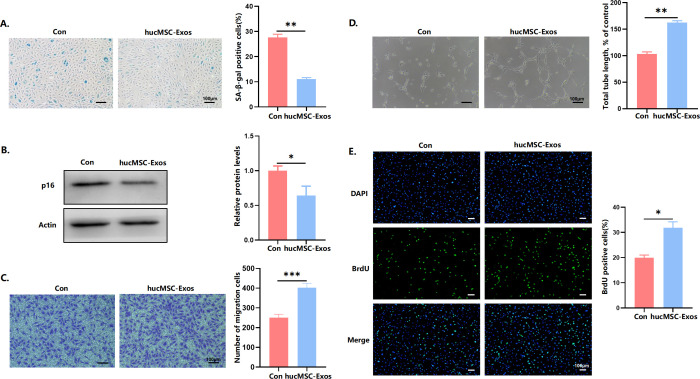
hucMSC-Exos delayed senescence, promoted proliferation, and increased angiogenic activity of endothelial cells. (A) Representative images of SA‐*β*‐gal staining of endothelial cells and percentages of SA‐*β*‐gal‐positive cells. Scale bars = 100 μm. (B) Quantitative analysis of P16 protein levels by western blot. (C) Representative micrographs and quantification of migrating cells in the transwell migration assay. Scale bars = 100 μm. (D) Representative images of the tube formation assay and quantitative analyses of the total tube length. Scale bars = 100 μm. (E) Representative BrdU staining images and quantification of BrdU‐positive cells as percentage of total cells. Data are presented as mean ± SD. Scale bars = 100 μm. *p < 0.05, **p < 0.01, ***p < 0.001.

Senescent endothelial cells were treated with hucMSC-Exos to study their impact on endothelial senescence. Treatment with hucMSC-Exos reduced the proportion of cells positive for SA-*β* (Fig 2A). According to western blot analysis, there was a notable downregulation in the expression levels of senescence marker p16 following hucMSC-Exos treatment (Fig 2B). Next, we studied the effect of hucMSC-Exos on endothelial functions. HucMSC-Exos treatment remarkably enhanced the migratory capacity of senescent endothelial cells, as demonstrated by transwell assay (Fig 2C). In addition, hucMSC-Exos treatment also restored the tube forming ability of senescent endothelial cells (Fig 2D). The BrdU assay results displayed a marked increase in the proliferation of endothelial cells when co-cultured with hucMSC-Exos (Fig 2E). Altogether, our findings showed that hucMSC-Exos could have a inhibitory effect against endothelial senescence.

### MiR-143-3p was abundant in hucMSC-Exos and delivered to endothelial cells

**Fig 3:**
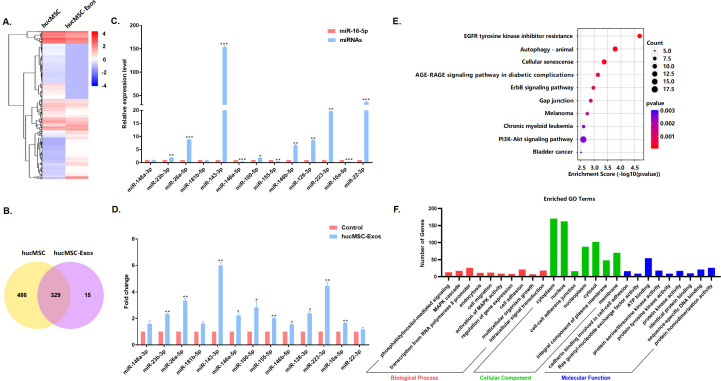
MiR-143-3p was abundant in hucMSC-Exos and delivered to endothelial cells. (A) Heatmap of differentially expressed microRNAs between hucMSCs and hucMSC-Exos. (B) Venn diagram of miRNAs of hucMSCs and hucMSC-Exos. (C) RT-qPCR verification of the most highly expressed and age-related miRNAs in hucMSC-Exos, using miR-16-5p with low expression in hucMSC-Exos as an external control. (D) The detection of the most highly expressed and age-related miRNAs in endothelial cells after treatment with hucMSC-Exos by RT-qPCR. (E) KEGG Enriched pathways of miR-143-3p predicted target genes. (F) GO analysis of miR-143-3p predicted target genes. Data are presented as mean ± SD. *p < 0.05, **p < 0.01, ***p < 0.001.

Exosomes contain a high abundance of miRNAs that play crucial roles in intercellular communication [17]. To further identify which miRNAs in hucMSC-Exos are responsible for prevention of endothelial cellular senescence, next-generation sequencing was applied to characterize miRNAs from hucMSCs and hucMSC-Exos, revealing the co-expression of 329 miRNAs (Fig 3A and 3B). Additionally, the expression levels of the most highly expressed and age-related miRNAs in hucMSC-Exos were validated using RT-qPCR based on the sequencing results. The experimental results confirmed that miR-143-3p had a high abundance in hucMSC-Exos, which was in line with the sequencing data (Fig 3C). Subsequently, RT-qPCR was employed to examine the levels of the above miRNAs in hucMSC-Exos-treated senescent endothelial cells. The results showed that miR-143-3p level of endothelial cells treated with hucMSC-Exos was markedly upregulated compared to the control group, suggesting a potential role of hucMSC-Exos-miR-143-3p in the regulation of endothelial function (Fig 3D). Furthermore, a miRNA target gene prediction website was utilized to forecast the miR-143-3p target genes. According to KEGG pathway analysis, miR-143-3p target genes were primarily enriched in EGFR tyrosine kinase inhibitor resistance, autophagy, cellular senescence, etc. (Fig 3E). GO analysis revealed the involvement of the predicted target genes in the biological process, cellular component and molecular function (Fig 3F). Most aforementioned signaling pathways are intricately interconnected with the aging process. These findings suggested that miR-143-3p contained in hucMSC-Exos could serve as a pivotal molecule for regulating endothelial senescence.

### MiR-143-3p alleviated senescence in endothelial cells

**Fig 4:**
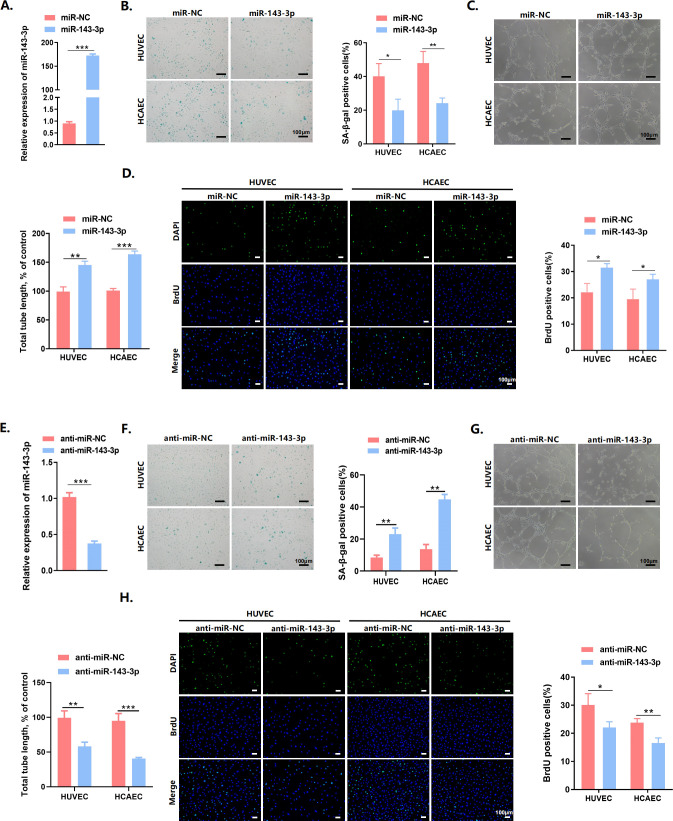
MiR-143-3p alleviated senescence in endothelial cells. (A) RT-qPCR analysis of miR-143-3p levels in young and senescent endothelial cells. (B) Representative images of SA‐*β*‐gal staining of endothelial cells and percentages of SA‐*β*‐gal‐positive cells in anti‐miR‐143‐3p- or anti‐miR‐NC-transfected endothelial cells. Scale bars = 100 μm. (C) Representative micrographs and statistical summary of in vitro Matrigel assays in anti‐miR‐143‐3p- or anti‐miR‐NC-transfected endothelial cells. Scale bars = 100 μm. (D) Representative BrdU staining images of endothelial cells and quantification of BrdU‐positive cells as percentage of total cells in anti‐miR‐143‐3p- or anti‐miR‐NC-transfected endothelial cells. Scale bars = 100 μm. (E) RT-qPCR analysis of miR-143-3p RNA expression in endothelial cells after transfection with miR-143-3p or miR-NC. (F) Representative images of SA‐*β*‐gal staining of endothelial cells and percentages of SA‐*β*‐gal‐positive cells in miR-143-3p- or miR-NC-transfected endothelial cells. Scale bars = 100 μm. (G) Representative micrographs and statistical summary of in vitro Matrigel assays in miR-143-3p- or miR-NC-transfected endothelial cells. Scale bars = 100 μm. (H) Representative BrdU staining images of endothelial cells and quantification of BrdU-positive cells as percentage of total cells in miR-143-3p- or miR-NC-transfected endothelial cells. Scale bars = 100 μm. Data are presented as mean ± SD. *p < 0.05, **p < 0.01, ***p < 0.001.

To explore the role of miR-143-3p in endothelial senescence, qPCR analysis was conducted to examine miR-143-3p levels in both young and senescent endothelial cells. The result showed that miR-143-3p expression declined with aging in endothelial cells (Fig 4A). Knockdown of miR‐143‐3p increased the percentage of SA‐*β*‐gal‐positive cells, decreased proliferation, and reduced the tube forming capacity of endothelial cells (Fig 4B–4D). Conversely, miR-143-3p overexpression exhibited opposite effects in endothelial cells (Fig 4E–4H). These results underscored the significance of hucMSC-Exos-miR-143-3p as a key molecule to delay endothelial senescence.

### COX-2 knockdown delayed endothelial cell senescence

**Fig 5:**
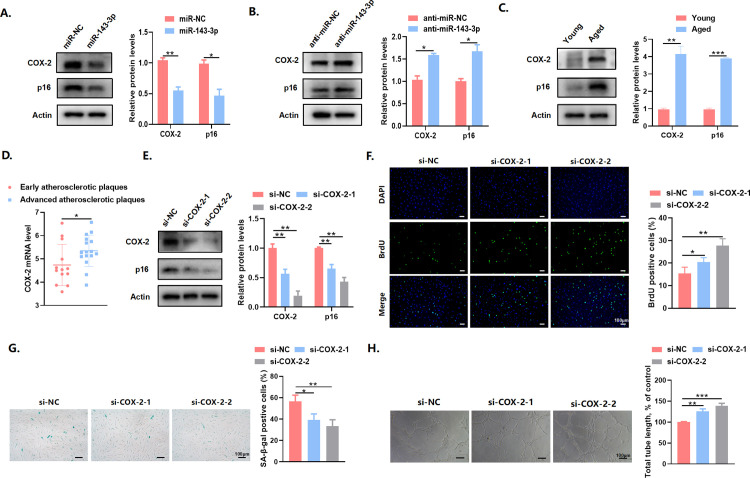
COX-2 knockdown delayed endothelial cell senescence. (A) COX-2, p16 protein expression and intensity ratio between COX-2, p16, and *β*-actin in endothelial cells transfected with miR-143-3p or miR-NC. (B) COX-2, p16 protein expression and intensity ratio between COX-2, p16, and *β*-actin in endothelial cells transfected with anti-miR-143-3p or anti-miR-NC. (C) Young and senescent endothelial cells were extracted, and levels of COX-2 and p16 in the extracts were determined by western blot. (D) Analysis of COX-2 mRNA level in early and advanced atherosclerotic plaques. (E) Western blot analysis of the levels of COX-2 and p16 in endothelial cells transfected with si-NC, si-COX-2-1, and si-COX-2-2. (F) BrdU assay, (G) SA-*β*-gal staining and (H) angiogenesis assay in endothelial cells after transfection with si-NC or si-COX-2-1/2. Scale bars = 100 μm. Data are presented as mean ± SD. *p < 0.05, **p < 0.01, ***p < 0.001.

MiRNAs are widely recognized for their ability to negatively regulate gene expression by either facilitating the degradation of target mRNAs or blocking translation [18]. Reports indicate that miR-143-3p targets COX-2 [19,20], and COX-2 expression increases with aging in most tissues partly because of reactive oxygen species (ROS), chemical reactions, physical shearing, and dietary molecules [21]. We also confirmed the negative regulatory effect of miR-143-3p on the protein expression of COX-2 and the senescence marker p16 in endothelial cells (Fig 5A). Conversely, when miR-143-3p levels were downregulated, there was an increased protein levels of COX-2 and p16 (Fig 5B). Furthermore, compared to young endothelial cells, there was an apparent decrease in COX-2 protein levels in senescent endothelial cells (Fig 5C), suggesting that COX-2 may be a significant regulator for endothelial senescence. We downloaded the gene expression profile of atherosclerotic samples from the Gene Expression Omnibus (GEO) database with an accession number of GSE28829. The analysis revealed higher levels of COX-2 expression in advanced atherosclerotic plaques compared to early atherosclerotic plaques (Fig 5D). As expected, knocking down COX-2 using siRNA remarkably downregulated the protein expression of p16 in endothelial cells, delayed cellular senescence, promoted cell proliferation and tube formation (Fig 5E–5H). The above results indicated that knockdown of COX-2 could inhibit endothelial senescence.

### COX-2 suppression alleviated the senescent phenotypes induced by miR-143-3p knockdown

**Fig 6:**
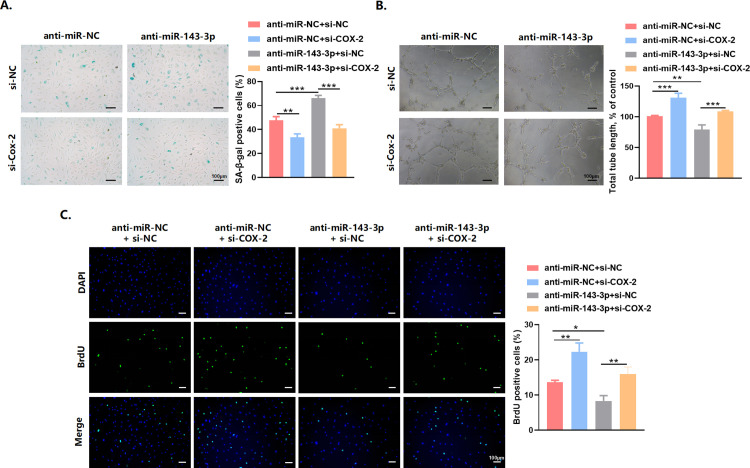
COX-2 suppression alleviated the senescent phenotypes induced by miR-143-3p knockdown. (A) β‐gal staining, (B) in vitro Matrigel assays, and (C) BrdU showed that miR-143-3p inhibitor could promote endothelial cell senescence, impair the tube formation ability and inhibit cell proliferation of endothelial cells. Knockdown of COX-2 could rescue these phenotypes. Scale bars = 100 μm. Data are presented as mean ± SD. *p < 0.05, **p < 0.01, ***p < 0.001.

In order to elucidate the interaction between miR-143-3p and COX-2 in regulating cell senescence, we performed transfection experiments on endothelial cells using miR-143-3p inhibitor and si-COX-2. The findings revealed that the downregulation of miR-143-3p markedly raised the proportion of cells positive for SA-*β*, which could be reversed by si-COX-2 (Fig 6A). Likewise, silencing of COX-2 could attenuate the effects of miR-143-3p inhibitor on endothelial proliferation and tubule formation (Fig 6B and 6C). In conclusion, these data suggested that miR-143-3p delayed endothelial senescence by targeting COX-2.

## Discussion

Recent literature on cellular senescence has considerably grown over the years, shedding light on the complex nature of senescent endothelial cells. It is now recognized that these cells are highly active, secretory and pro-inflammatory, and exhibit an aberrant morphological phenotype [22]. Furthermore, it has been convincingly demonstrated that endothelial senescence significantly contributes to an array of cardiovascular and metabolic diseases [23]. Therefore, delaying endothelial senescence is an effective strategy to prevent age-related diseases. MSCs-derived exosomes offer a promising new approach for treating age-related diseases, potentially avoiding the typical risks and hurdles of traditional stem cell treatments. In the present study, we found that hucMSC-Exos effectively delay endothelial senescence. Further studies showed that the underlying molecular mechanism responsible for this effect is miR-143-3p, the most abundant miRNA in hucMSC-Exos, which targets COX-2 and downregulates its expression.

MSCs have numerous biological functions, including anti-inflammation, angiogenesis, and immune modulation, which lay the foundation for their therapeutic potential in age-related diseases [24]. However, stem cell transplantation is plagued by drawbacks like malignant transformation, cell rejection, and high cost [25]. Stem cell-derived exosomes can play similar biological roles to stem cells, providing a cell-free therapeutic approach as a substitute for conventional stem cell treatment. Chen and colleagues demonstrated that exosomes sourced from embryonic stem cells exhibited anti-aging property through the transfer of miR-200a to D-gal-induced senescent endothelial cells, subsequently triggering the activation of Nrf2 signaling pathway [26]. Xiao *et al.* identified that adipose mesenchymal stem cell-secreted extracellular vesicles acted as a nanotherapeutic agent to rejuvenate senescent endothelial cell induced by H_2_O_2_ through miR-146/Src pathway [27]. The effect of hucMSC-Exos in a replicative endothelial senescence model, along with associated mechanisms, remains elusive. In this research, we isolated hucMSC-Exos and confirmed the ability of hucMSC-Exos to effectively ameliorate vascular endothelial cell senescence and restore the angiogenic function. Based on our and other results, hucMSC-Exos may be anti-aging therapeutic agents, and their therapeutic effects should be studied more extensively.

Exosomes play a crucial role as mediators of intercellular communication by transporting miRNAs to recipient cells [28]. MiRNAs are small noncoding RNAs that modulate gene expression through pairing with complementary sequences in the 3′ untranslated region (UTR) of target mRNAs, thereby mediating their degradation [29]. Exosomal miRNAs are involved in regulating endothelial senescence. Exosomes secreted by endothelial cells suppressed the expression of the ataxia telangiectasia mutation (ATM) in recipient cells through a mir-214-dependent mechanism, thus delaying endothelial senescence while enhancing migration and angiogenesis [30]. Senescent osteoblast-derived exosomes could speed up the aging process of endothelial cells and impede their proliferation and migration, facilitated by miR-214-3p/L1CAM pathway [31]. Our results showed that hucMSC-Exos is enriched with miR-143-3p, which exhibited increased levels in endothelial cells after treatment with hucMSC-Exos. Subsequent study revealed a decline in the levels of miR-143-3p in senescent endothelial cells. Upregulation of miR-143-3p delayed endothelial senescence while enhancing proliferation and angiogenesis. Conversely, downregulating miR-143-3p showed the opposite influence. Interestingly, studies investigating the impact of miR-143-3p on cellular senescence yielded conflicting results. It is reported that the expression of miR-143-3p decreased in satellite cells with age, correlating with elevated expression levels of its target, Igfbp5 [32]. A previous study showed that miR-143-3p overexpression promoted the proliferation and migration of adipose-derived stem cells (ASCs) while ameliorating cellular senescence in ASCs [33]. However, a study observed significantly reduced viability, invasion, and migration of hypoxia/reoxygenation-damaged cardiac microvascular endothelial cells after overexpressing miR-143-3p [34]. We cannot exclude the possibility that the varying outcomes regarding the impact of miR-143-3p on cellular senescence may be attributed to different cell lines, strains and diverse cell culture methods. As far as we are aware, there exists no investigation into the impact of hucMSC-Exos-miR-143-3p on endothelial cell senescence. Altogether, our research suggested that miR-143-3p may be a novel target for the treatment of age-related diseases via rescue senescent endothelial cells.

Furthermore, COX-2 was identified as a potential target gene for miR-143-3p according to TargetScan bioinformatics prediction. Previous findings consistently suggested that COX-2 is a downstream target gene of miR-143-3p, which suppresses COX-2 by directly binding to its 3′-UTR [35,36]. COX-2 is a prostaglandin synthase that converts arachidonic acid to prostaglandin H2 (PGH2) [37]. Typically, COX-2 remains unexpressed in most cells under normal circumstances, but its levels rise significantly following inflammation to exacerbate endothelial dysfunction [38]. Evidence suggested a pivotal function of COX-2 in endothelium-dependent contraction and its highly expressed in aged hamsters [39]. Several studies have reported that the inhibition of COX-2 could improve endothelial function in individuals suffering from coronary artery disease and hypertension [40,41]. Our study demonstrated the elevation of COX-2 levels in aged endothelial cells and knockdown of COX-2 ameliorated endothelial senescence; suppression of COX-2 could alleviate the senescent phenotypes caused by miR-143-3p knockdown. These results highlighted the significant role of miR-143-3p in the hucMSC-Exos-induced modulation of endothelial senescence by suppressing its target COX-2.

Collectively, the present study showed that hucMSC-Exos exerted anti-aging effects by transporting their encapsulated miR-143-3p into senescent endothelial cells to silence COX-2 expression. Hence, miR-143-3p could potentially be used as a promising target for preventing endothelial cell senescence and its correlated vascular complications. These findings offer novel insights and cell-free therapies for delaying senescence of vascular endothelial cells.

## Supporting information

S1 File S1 Fig. Identification of human umbilical cord mesenchymal stem cells. (A) Morphology of hucMSCs (passages 1 and 3). Scale bars: 500 μm (left); 100 μm (right). (B) Oil red O staining for lipogenic induced differentiation of hucMSCs and alizarin red staining for osteogenic induced differentiation of hucMSCs. Scale bars: 500 μm (left); 100 μm (right). (C) Flow cytometry to characterize the phenotype of hucMSCs. Isotype control is shown in blue and the experimental group is shown in blue. S1 Table. Primers and oligomers used in this study.
(DOCX)

S2 File Supplementary Figures S2–S7. (PDF)

## Abbreviations

MSCs: Mesenchymal stem cells
hucMSCs: Human umbilical cord mesenchymal stem cells
hucMSC-Exos: Exosomes derived from human umbilical cord mesenchymal stem cells
COX-2: Cyclooxygenase-2
HCAECs: Human coronary artery endothelial Cells
ECM: Endothelial cell medium
PDL: Population doubling level
TEM: Transmission electron microscopy
NTA: Nanoparticle tracking analysis
ROS: Reactive oxygen species
ATM: Ataxia telangiectasia mutation
ASCs: Adipose-derived stem cells
PGH2: Prostaglandin H2
