# Supplementary material for: Umbilical cord mesenchymal stem cell exosomal miR-143-3p delays endothelial cell senescence through targeting COX-2
Source: PLoS One. 2025 Jul 11;20(7):e0327173. doi: 10.1371/journal.pone.0327173 (PMC12250453; doi:10.1371/journal.pone.0327173)
Supplement: S1 File — (A) Morphology of hucMSCs (passages 1 and 3). Scale bars: 500 μm (left); 100 μm (right). (B) Oil red O staining for lipogenic induced differentiation of hucMSCs and alizarin red staining for osteogenic induced differentiation of hucMSCs. Scale bars: 500 μm (left); 100 μm (right). (C) Flow cytometry to characterize the phenotype of hucMSCs. Isotype control is shown in blue and the experimental group is shown in blue. S1 Table. Primers and oligomers used in this study. (DOCX) [file pone.0327173.s001.docx]

**Supporting Information**

**S1 Fig. Identification of human umbilical cord mesenchymal stem cells.** (A) Morphology of hucMSCs (passages 1 and 3). Scale bars: 500 μm (left); 100 μm (right). (B) Oil red O staining for lipogenic induced differentiation of hucMSCs and alizarin red staining for osteogenic induced differentiation of hucMSCs. Scale bars: 500 μm (left); 100 μm (right). (C) Flow cytometry to characterize the phenotype of hucMSCs. Isotype control is shown in blue and the experimental group is shown in blue.


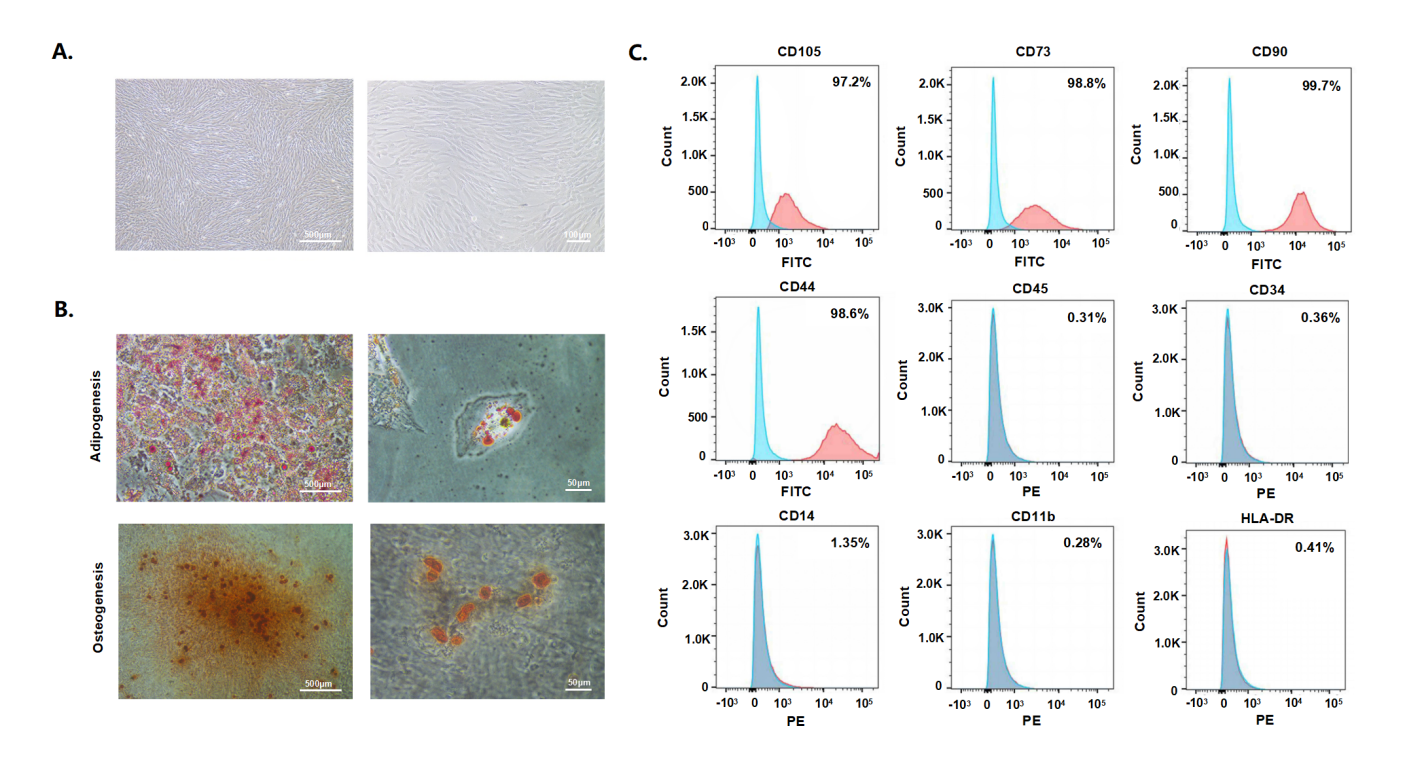


**S1 Table . Primers and oligomers used in this study**

|  | **Name** | **Sense（5'-3'）** | **Antisense（5'-3'）** |
| --- | --- | --- | --- |
| Primer | U6 | CTCGCTTCGGCAGCACA | AACGCTTCACGAATTTGCGT |
|  | miR-143-3p | CGCGTGAGATGAAGCACTG | AGTGCAGGGTCCGAGGTATT |
| Oligomer | miR-NC | UUCUCCGAACGUGUCACGUTT | ACGUGACACGUUCGGAGAATT |
|  | miR-143-3p mimic | UGAGAUGAAGCACUGUAGCUC | GCUACAGUGCUUCAUCUCAUU |
|  | anti-NC | CAGUACUUUUGUGUAGUACAA |  |
|  | miR-143-3p inhibitor | GAGCUACAGUGCUUCAUCUCA |  |
