# Supplementary material for: Umbilical cord mesenchymal stem cell exosomal miR-143-3p delays endothelial cell senescence through targeting COX-2
Source: PLoS One. 2025 Jul 11;20(7):e0327173. doi: 10.1371/journal.pone.0327173 (PMC12250453; doi:10.1371/journal.pone.0327173)

Supplementary data

Supplementary Fig. S2. Exosome protein markers expression in hucMSCs and hucMSC-Exos.

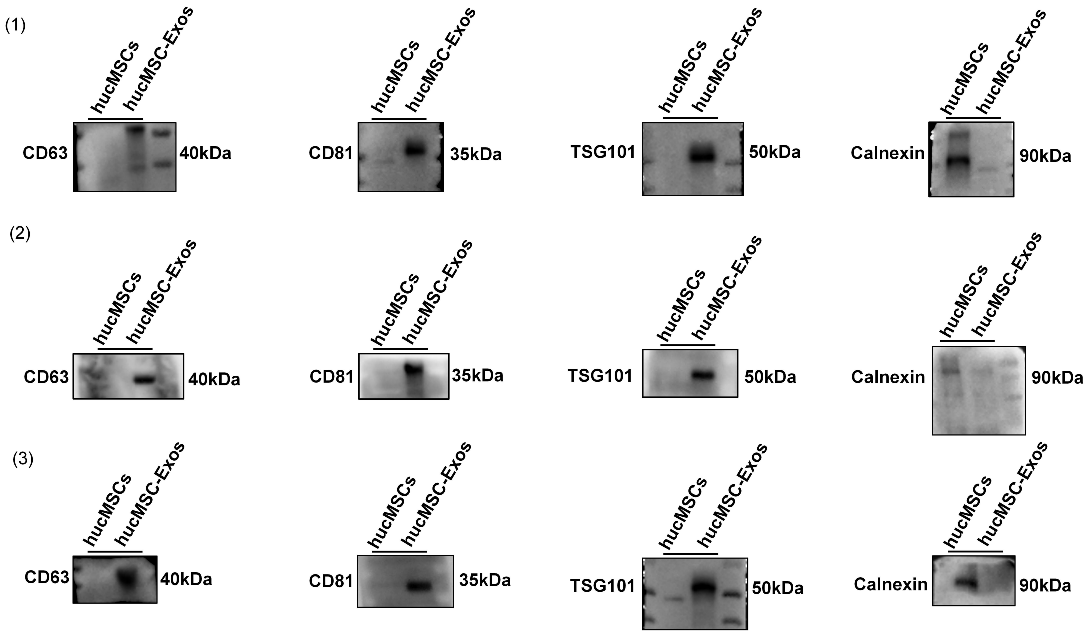

Supplementary Fig. S3. p16 protein expression in endothelial cells treated with hucMSC-Exos.

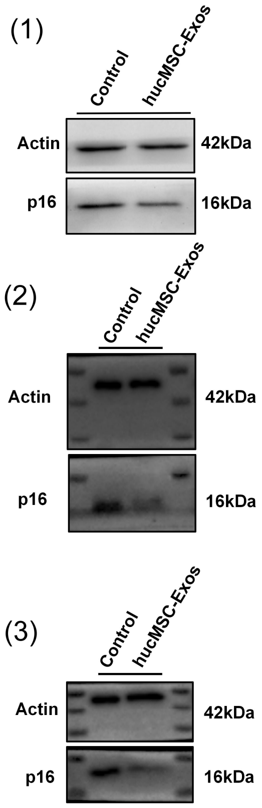

Supplementary Fig. S4. The expression levels of COX-2 and p16 in endothelial cells transfected with miR-143-3p or miR-NC.

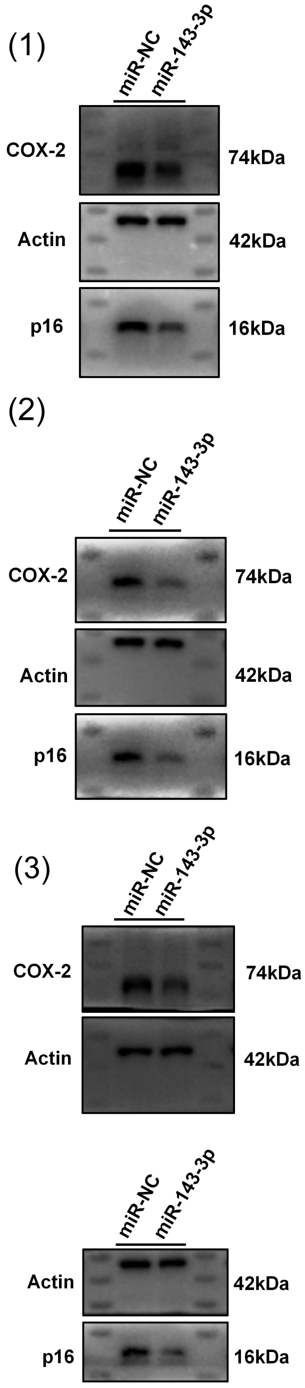

Supplementary Fig. S5. The expression levels of COX-2 and p16 in endothelial cells transfected with anti-miR-143-3p or anti-miR-NC.

(1)

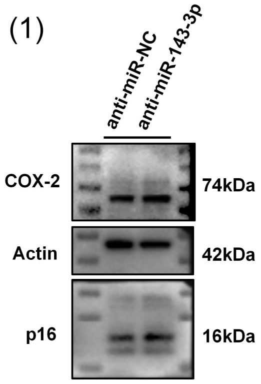

(2)

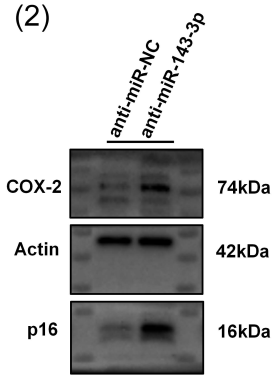

(3)

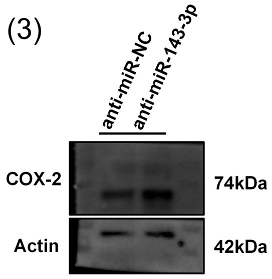

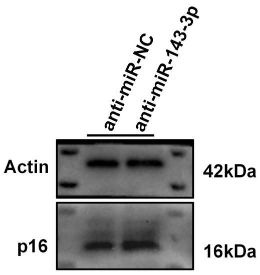

Supplementary Fig. S6. The expression levels of COX-2 and p16 in young and senescent endothelial cells.

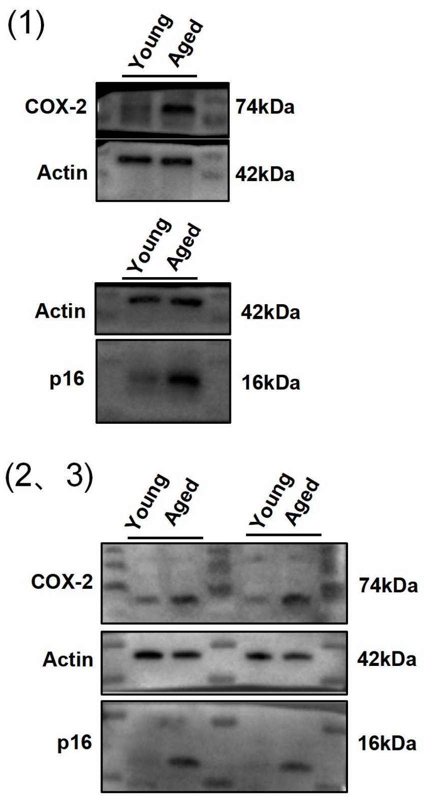

Supplementary Fig. S7. The expression levels of COX-2 and p16 in endothelial cells transfected with si-NC, si-COX-2-1, and si-COX-2-2.

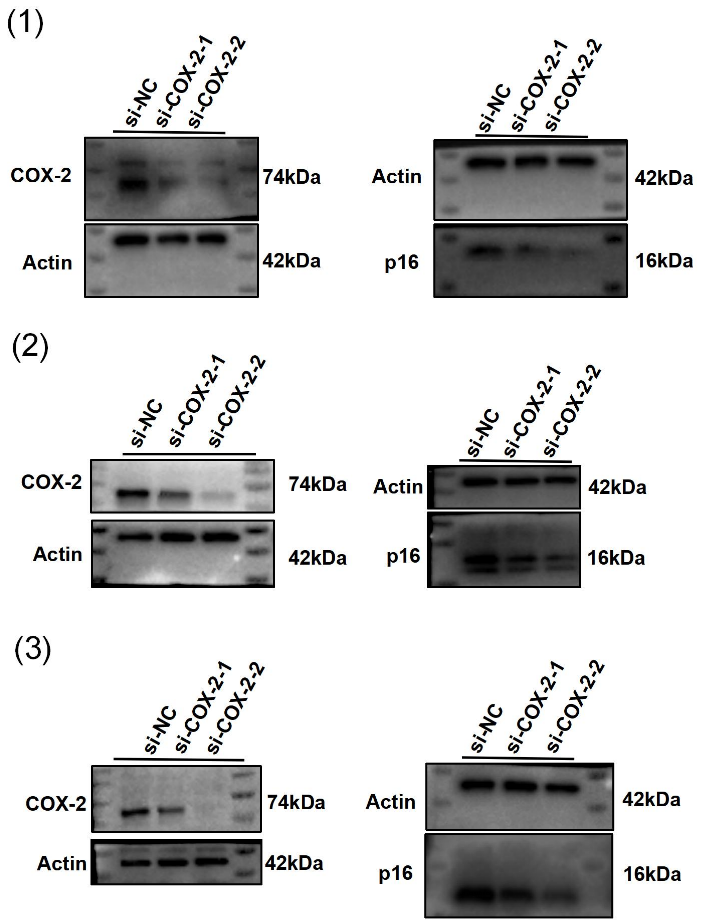

Supplement: S2 File — (PDF) [file pone.0327173.s002.pdf]
